# One-Year Experience With the New Kidney Allocation Policy at a Single Center and an OPO in the Midwestern United States

**DOI:** 10.3389/ti.2022.10798

**Published:** 2022-12-07

**Authors:** Tarek Alhamad, Gary Marklin, Mengmeng Ji, Richard Rothweiler, Su-Hsin Chang, Jason Wellen

**Affiliations:** ^1^ Division of Nephrology, School of Medicine, Washington University in St. Louis, St. Louis, MO, United States; ^2^ Mid-America Transplant Organ Procurement Organization, St. Louis, MO, United States; ^3^ Division of Public Health Sciences, Department of Surgery, School of Medicine, Washington University in St. Louis, St. Louis, MO, United States; ^4^ Department of Surgery, School of Medicine, Washington University in St. Louis, St. Louis, MO, United States

**Keywords:** discard, kidney allocation, OPO, import kidneys, policy

Dear Editors,

The new kidney allocation policy implemented in March 2021 has replaced the traditional donation service areas (DSAs) boundaries with a single 250-nautical mile circle centered around the donor hospital to decrease geographic disparities in waiting time for deceased donor kidney transplantation (DDKT) (1). Despite the extensive discussion about the policy development and simulation models for potential consequences (2–4), few studies have quantitatively investigated the practical impacts of this redistricting change on transplant center-level and organ procurement organization (OPO)-level practices. An early evaluation of a large rural transplantation program in the East Coast found that the new kidney allocation policy has led to an increase in Kidney Donor Profile Index (KDPI) of donors with longer cold ischemia time (CIT), leading to higher delayed graft function (DGF) rates (5). As a large transplant center located in the Midwestern United States, in this study, we evaluate the impacts of the new allocation policy on our transplant center and its OPO, Mid-American Transplant.

This is a retrospective, cross-sectional analysis of organ offers, allograft outcomes, and attributed costs before and after the change of allocation system. The data from our single transplant center and its OPO between 15 March 2019 and 14 March 2022 was analyzed for three time periods, i.e., pre-allocation era without pandemic (15 March 2019 to 14 March 2020), pre-allocation era with pandemic (15 March 2020 to 14 March 2021), and post-allocation era with pandemic (15 March 2021 to 14 March 2022). For all pre- and post-allocation comparations, data of pre-allocation era with pandemic was used to adjust for the potential impacts of the pandemic.

There were 254, 234, and 224 DDKT performed in our transplant center during three time periods, respectively. No statistically significant difference was found regarding the percentage of imported kidneys, DGF, CIT, and KDPI due to the pandemic (Figure 1). Compared to the pre-allocation era with pandemic, the percentage of imported kidneys has increased from 14% to 60% (*p* < 0.001) in the post-allocation era; the percentage of DGF has increased from 21% to 30% (*p* < 0.05). The CIT has increased from an average of 15 h to 20 h (*p* < 0.001). The KDPI has increased from an average of 40% to 50%, with the percentage of KDPI ≥85% increased from 6% to 12% (*p* < 0.001). While the number of transplants performed did not increase, the number of organ offers became extremely voluminous and heavily impacted our ability to perform surgeries the next day after being awake all night reviewing those organ offers. As a result of increased workload and dramatic increase in donors offered in the night during the post-allocation era, our transplant center added 4 new Full-time Equivalent (FTE) positions, with 1 FTE on thoracic offers and 1 FTE on abdominal offers for 24 h periods and having 24 h off.

**FIGURE 1:**
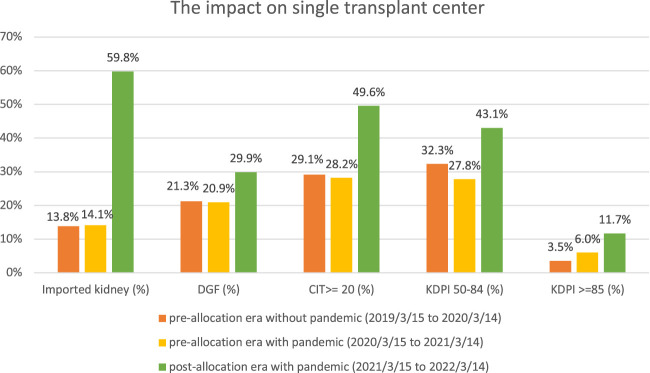
Comparison of the pre- and post-new kidney allocation policy in the kidney transplant center.

For the OPO, the average sequence number for all kidneys accepted for three time periods was 759, 534, and 1,491, respectively. This dramatic increase was driven by expedited kidney allocation, which was a response to the significant decline in kidney utilization and increased discards experienced in the post-allocation period. The number of kidneys exported also increased from 134 in pre-allocation era to 261 in the post-allocation era. In anticipation of increased offers and increased import organs, the OPO hired one additional Organ Import Coordinator (OIC) and one additional Organ Recovery Coordinator (ORC). The OIC handles the incoming organ offers for the transplant centers and assists with planning and logistics. The ORC is the preservationist, who also cannulates and pumps imported kidneys and monitors them for a while before sending them to the transplant center. Additionally, compared to the pre-allocation era with pandemic, the percentage of imported kidneys increased from 10% to 32% in the post-allocation era (*p* < 0.001). As the percentage of imported kidney increases, the cost of kidneys increases accordingly. The cost of transportation of a local donor to a local transplant center was $60 or less, whereas it takes between $600 and $1500, on average, when shipping a kidney across the country. For local kidneys, CIT increased from an average of 16 h to 19 h (*p* < 0.001); the percentage of pumped kidneys decreased from 60% to 52% (*p* < 0.05).

Our analyses show that the implementation of new kidney allocation policy has posed an additional operational and financial burden to our transplant center and its local OPO. Our results were consistent with the findings of Rohan et al. (5) and the anticipations about the complexity and unintended detrimental consequences of the new kidney allocation (6, 7). While this single transplant center analysis needs to be interpreted carefully, it remains unknown if these changes would continue to be the new norm or would regress after reaching a new equilibrium. Continuous monitoring the efficiency and evaluating the impacts of the new allocation policy in different regions in the United States are warranted.

## Data Availability

The original contributions presented in the study are included in the article/supplementary material, further inquiries can be directed to the corresponding author.

## Abbreviations

DSAs, donation service areas; OPO, organ procurement organization; DGF, delayed graft function; CIT, cold ischemia time; KDPI, kidney donor profile index.
